# Supplementary material for: The Inflammasome-Dependent Dysfunction and Death of Retinal Ganglion Cells after Repetitive Intraocular Pressure Spikes
Source: Cells. 2023 Nov 15;12(22):2626. doi: 10.3390/cells12222626 (PMC10670000; doi:10.3390/cells12222626)
Supplement: Supplementary file 1 [file cells-12-02626-s001.zip › cells-2660191-supplementary.pdf]

Supplement Table S1. Real-time PCR gene-specific primer pairs

Gene	Forward	Reverse
Gsdmd	TCATGTGTCAACCTGTCAATCAAGGACAT	CATCGACGACATCAGAGACTTTGAAGGA
Casp1	CTGACAAGATCCTGAGGGCA	AAAGATTTGGCTTGCCTGGG
Casp3	AAGGAGCAGCTTTGTGTGTGT	AAGAGTTTCGGCTTTCCAGTC
Casp8	CTCCGAAAAATGAAGGACAGA	CGTGGGATAGGATACAGCAGA
Aim2	AAGTGCGAGGAAGGAGACAA	TTCAAGGAGCAGCATCAGGA
Nlrp1	ATAGAGGAGCAGGCAGGTCT	CGTGCTCCTGGAAAGGTTCT
Nlrp3	GCTCCAACCATTCTCTGACC	AAGTAAGGCCGGAATTCACC
Gfap	GGTTGAATCGCTGGAGGAG	CTGTGAGGTCTGGCTTGG
Aif1/Iba1	AGAGAGGTGTCCAGTGGC	CCCCACCGTGTGACCTCC
Il1b	GACCTTCCAGGATGAGGACA	AGGCCACAGGTATTTTGTCG
Tnfa	CAAAATTCGAGTGACAAGCCTG	GAGATCCATGCCGTTGGC

Supplemental Table S2. Primary Antibodies

Antibodies	Manufacture	Reference
HIF1α	Santa Cruz, sc-13515, mouse mAb	PMID: 18658046 ǀ Transcriptional activation of HIF-1 by RORalpha and its role in hypoxia signaling. ǀ Kim, E.J. et al. 2008. Arterioscler Thromb Vasc Biol. 28: 1796-1802.<br>PMID: 18178340 ǀ Hypoxia induces class III beta-tubulin gene expression by HIF-1alpha binding to its 3′ flanking region. ǀ Gene. 409: 100-8.<br>PMID: 36714464 ǀ Ochratoxin A induces endoplasmic reticulum stress and fibrosis in the kidney via the HIF-1α/miR-155-5p link. ǀ Yang, SA. et al. 2023. Toxicol Rep. 10: 133-145.
RBPMS	GTX, 118619, rabbit pAb	PMID: 27450912 ǀ A mouse retinal explant model for use in studying neuroprotection in glaucoma. ǀ Exp Eye Res. 2016 Oct:151:38-44.<br>PMID: 32832230 ǀ A Minimally Invasive Experimental Model of Acute Ocular Hypertension with Acute Angle Closure Characteristics. ǀ Chong, RS. Et al. Transl Vis Sci Technol. 2020 Jun 22;9(7):24<br>PMID: 37029422 ǀ N,N-Dimethyl-3β-hydroxycholenamide attenuates neuronal death and retinal inflammation in retinal ischemia/reperfusion injury by inhibiting Ninjurin 1. ǀ Shi, Y. et al. J Neuroinflammation. 2023 Apr 7;20(1):91
GFAP	Sigma, C9205, mouse mAb	PMID33960512 ǀ Comparison of the effects of two therapeutic strategies based on olfactory ensheathing cell transplantation and repetitive magnetic stimulation after spinal cord injury in female mice. ǀ Delarue Q Et al. Journal of neuroscience research (2021-05-08)<br>PMID30661753 ǀ<br>Environmental Control of Astrocyte Pathogenic Activities in CNS Inflammation. ǀ Wheeler, MA. Et al. Cell (2019-01-22)<br>PMID21712024 ǀ Microglia/macrophages migrate through retinal epithelium barrier by a transcellular route in diabetic retinopathy: role of PKCζ in the Goto Kakizaki rat model. ǀ Omri, S. et al. The American journal of pathology (2011-06-30)

CASP1	Novus, 14F68 NB100-56565SS, mouse	PMID: 19221555 | The inflammasome: a caspase-1-activation platform that regulates immune responses and disease pathogenesis. | Franchi, L., et al. Nature immunology. (2009).<br>PMID: 32729154 | Molecular mechanisms activating the NAIP-NLRC4 inflammasome: Implications in infectious disease, autoinflammation, and cancer. | Kay, C., et al. Immunological reviews. (2020).<br>PMID: 25526085 | Old, new and emerging functions of caspases. | Shalini, S., et al. Cell death and differentiation. (2015)
CASP3/7	Cell Signaling, 9661S, rabbit	PMID: 35882943 | Exercise-induced FNDC5/irisin protects nucleus pulposus cells against senescence and apoptosis by activating autophagy. | Zhou, W. et al. Exp Mol Med. 2022 Jul;54(7):1038-1048<br>PMID: 35618762 | Temperature sensitivity of DNA double-strand break repair underpins heat-induced meiotic failure in mouse spermatogenesis. | Hirano, K. et al. Commun Biol . 2022 May 26;5(1):504<br>PMID: 35488301 | Macrophage-based delivery of interleukin-13 improves functional and histopathological outcomes following spinal cord injury. | Broeckhoven, JV. Et al. J Neuroinflammation. 2022 Apr 29;19(1):102
TNFα	R&D systems, AF-410-NA, goat	PMID: 19435787 | Cooperative phagocytes: resident microglia and bone marrow immigrants remove dead photoreceptors in retinal lesions. | Joly, S. et al. Am J Pathol. 2009 Jun;174(6):2310-23.<br>PMID: 28094236 | Deficiency of PTP1B Attenuates Hypothalamic Inflammation via Activation of the JAK2-STAT3 Pathway in Microglia. | Tsunekawa, T. et al. EBioMedicine. 2017 Feb:16:172-183.<br>PMID: 19780903 | Attenuation of AD-like neuropathology by harnessing peripheral immune cells: local elevation of IL-10 and MMP-9. | Koronyo-Hamaoui, M. et al. J Neurochem. 2009 Dec;111(6):1409-24.
IL1β	Cell Signaling, 8689S, rabbit	PMID: 30930743 | Inflammasome Activation Induces Pyroptosis in the Retina Exposed to Ocular Hypertension Injury. | Pronin, A. et al. Front Mol Neurosci. 2019 Mar 13:12:36.<br>PMID: 26461208 | P2RX7 purinoceptor: a therapeutic target for ameliorating the symptoms of duchenne muscular dystrophy. | Sinadinos, A. et al. PLoS Med. 2015 Oct 13;12(10):e1001888.
CD11b	ThermoFisher 12-0112-82	PMID: 25010693 | Neutrophils contribute to excess serum BAFF levels and promote CD4+ T cell and B cell responses in lupus-prone mice. | Coquery, CM. et al. PLoS One. 2014 Jul 10;9(7):e102284<br>PMID: 32086278 | Cellular and Molecular Mechanisms of Kidney Injury in 2,8-Dihydroxyadenine Nephropathy. | Klinkhammer, BM. Et al. J Am Soc Nephrol. 2020 Apr;31(4):799-816.<br>PMID: 19130474 | The cellular niche of Listeria monocytogenes infection changes rapidly in the spleen. | Aoshi, T. et al. Eur J Immunol. 2009 Feb;39(2):417-25.
CD45	ThermoFisher 14-0451-82, rat	PMID: 33510091 | VX-765 reduces neuroinflammation after spinal cord injury in mice. | Chen, J. et al. Neural Regen Res. 2021 Sep;16(9):1836-1847.<br>PMID: 32198351 | Therapeutic modulation of phagocytosis in glioblastoma can activate both innate and adaptive antitumour

		immunity. ǀ von Roemeling, CA. et al. Nat Commun. 2020 Mar 20;11(1):1508.<br>PMID: 31523027 ǀ Chronic Inflammation Directs an Olfactory Stem Cell Functional Switch from Neuroregeneration to Immune Defense. ǀ Chen, M. et al. Cell Stem Cell. 2019 Oct 3;25(4):501-513.e5.
CASP8	Novus, NB100-56116, rabbit	PMID: 28479336 ǀ Desiccating stress worsens alkali burn injury by magnifying caspase-8-induced imbalance of NLRP3 and NLRP6. ǀ Hua, X. et al. J Allergy Clin Immunol. 2017 Oct;140(4):1172-1176.e3.<br>PMID: 24315969 ǀ Topical interferon-gamma neutralization prevents conjunctival goblet cell loss in experimental murine dry eye. ǀ Zhang, X. et al. Exp Eye Res. 2014 Jan:118:117-24.<br>PMID: 32946645 ǀ Sterile inflammation drives multiple programmed cell death pathways in the gut. ǀ Ruera, CN. Et al. J Leukoc Biol. 2021 Jan;109(1):211-221
GSDMD	Abcam, 209845, rabbit pAb	PMID: 35892155 ǀ STING mediates neuroinflammatory response by activating NLRP3-related pyroptosis in severe traumatic brain injury. ǀ Zhang, L. et al. J Neurochem. 2022 Sep;162(5):444-462<br>PMID: 35780081 ǀ P2X7R/NLRP3 signaling pathway-mediated pyroptosis and neuroinflammation contributed to cognitive impairment in a mouse model of migraine. ǀ Wang, Y. et al. J Headache Pain. 2022 Jul 2;23(1):75.<br>PMID: 33656514 ǀ Vincristine-induced peripheral neuropathy is driven by canonical NLRP3 activation and IL-1β release. ǀ Starabova, H. et al. J Exp Med. 2021 May 3;218(5):e20201452

Supplement Figures

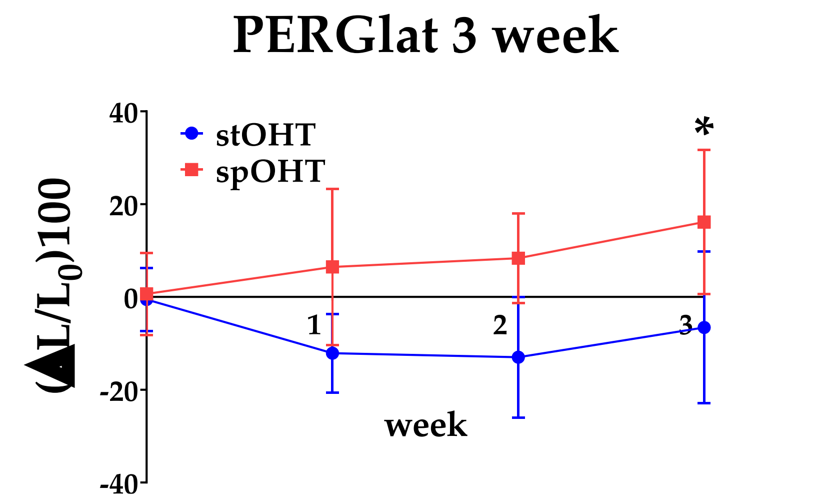

Supplemental Figure S1. Changes in PERG latency following the spOHT and stOHT challenges. Changes at 1 and 2 weeks were not statistically significant. Latency significantly increased at 3 weeks following spike. Mean ± SE, *P <0.05, n = 10-15.

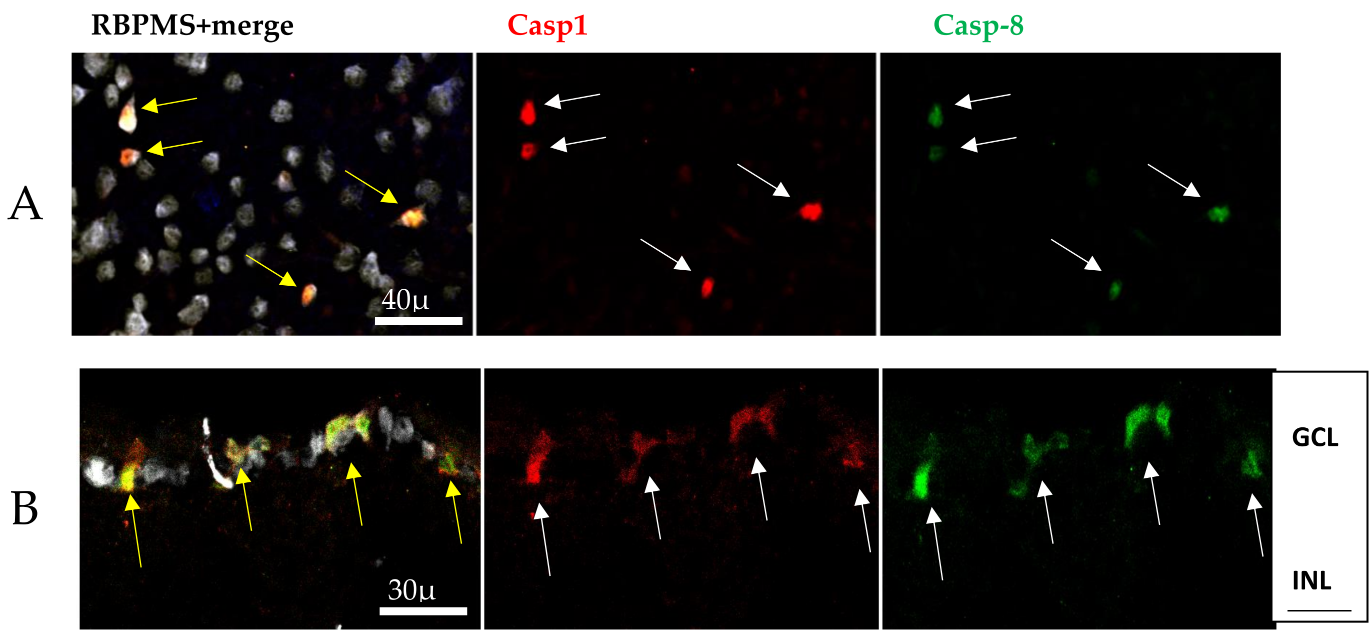

Supplement Figure S2. Co-activation of caspase-1 and caspase-8 in RGCs. Representative micrographs of retinal wholemounts (A) and cross sections (B) showing active CASP1 (red) and CASP8 (green) labeling assayed with FAM-FLICA dye-conjugated specific substrates. RGCs were labeled with antibodies against the RBPMS marker protein (white).

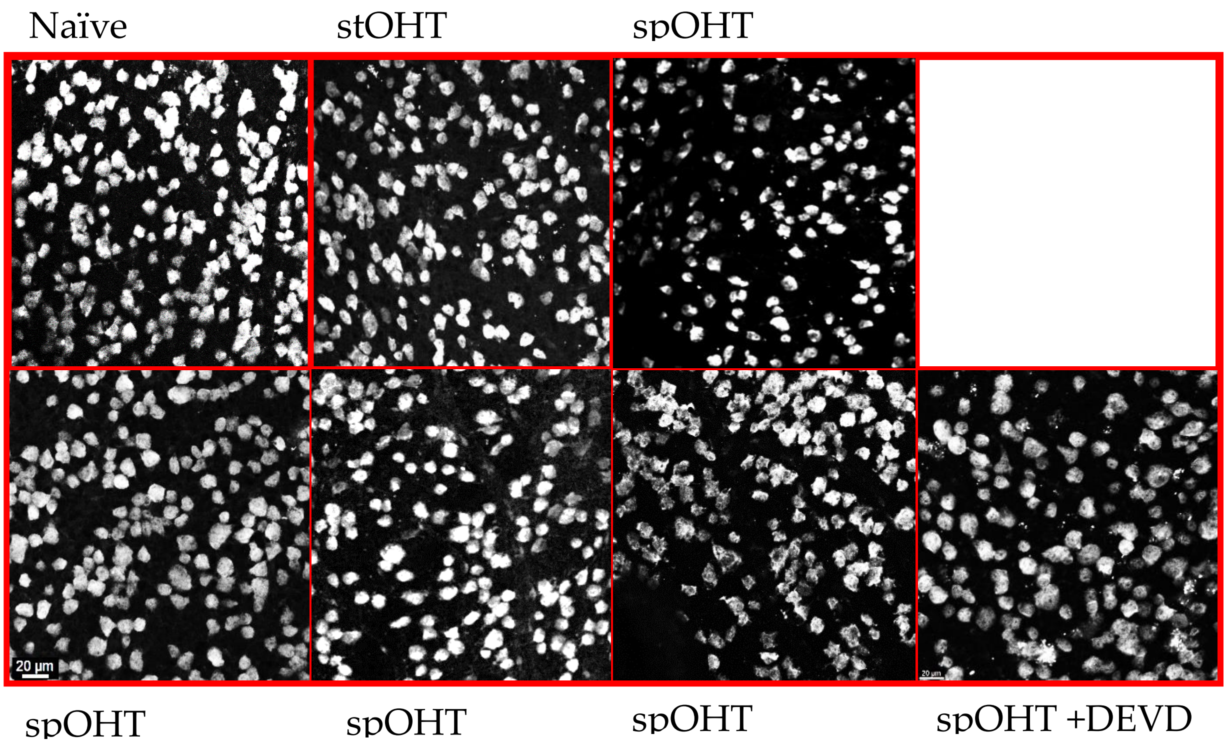

Supplement Figure S3. Representative micrographs of wholemount retinas stained for the RBPMS marker to assess RGC density at 7 dpi in the mid-retina exposed to either spOHT or stOHT challenges

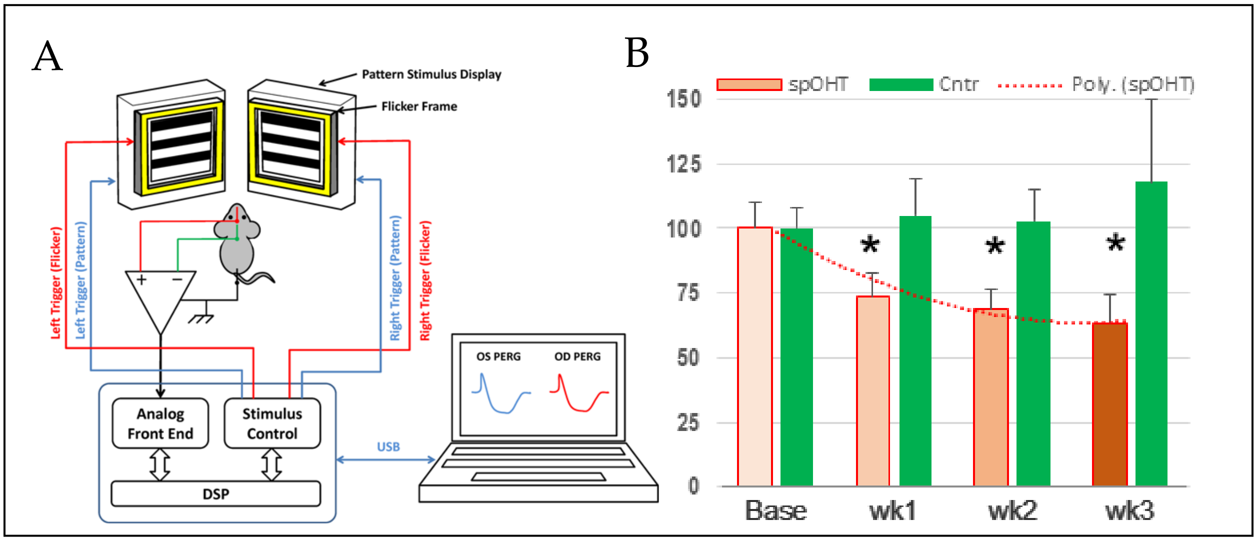

Supplement Figure S4. A. Diagram depicting rge experimental setup for a PERG recording experiment. **B**. PERG amplitude changes vs baseline at 1, 2 and 3 weeks after a challenge with a single episode of spOHT (7x1)

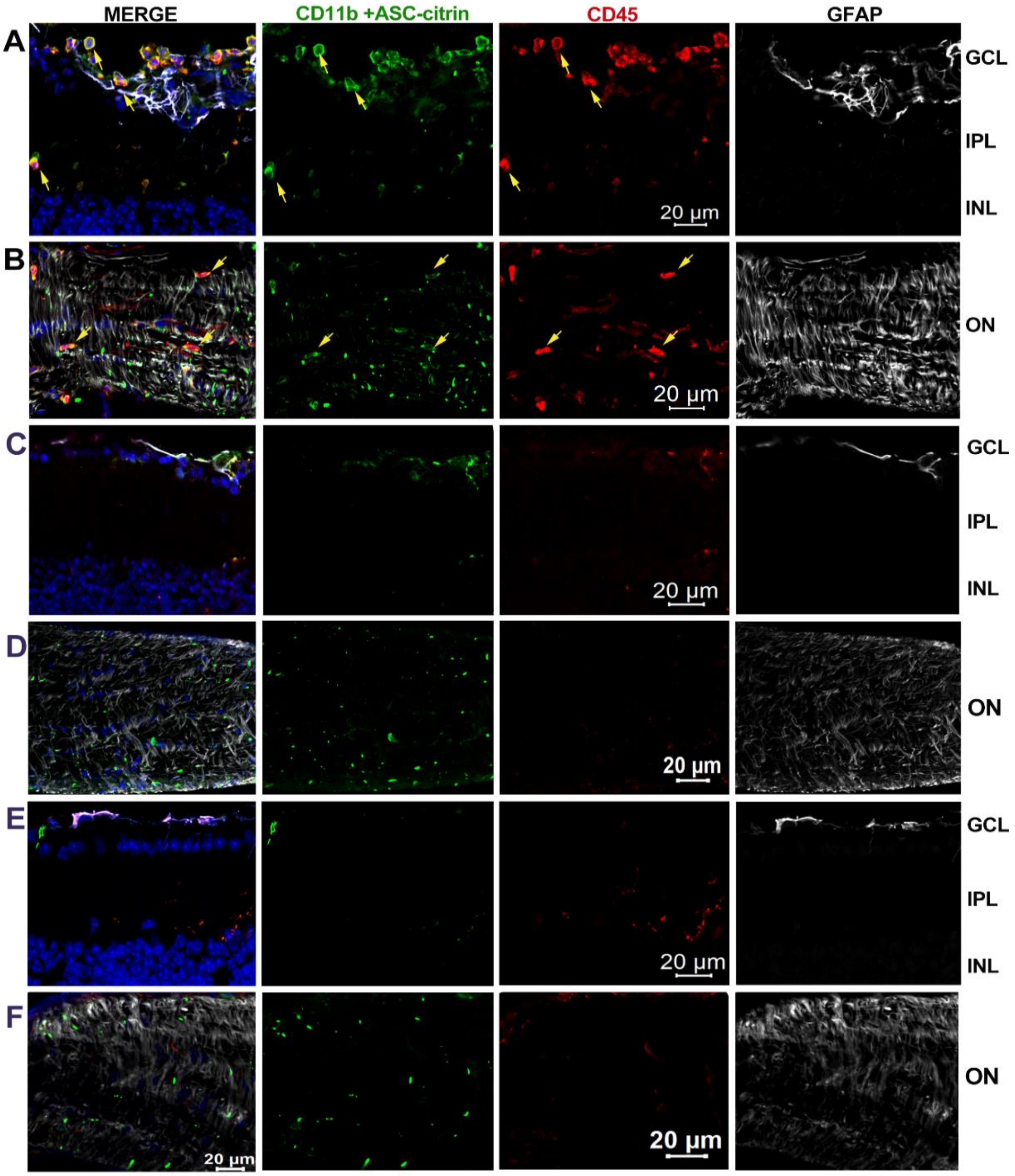

Supplement Figure S5 (full version of Fig. 9**). Monocyte/macrophages infiltration into the inner retina and optic nerves after spOHT challenge. A, B.** Representative micrographs for cells expressing CD45 (red) and CD11b (green) markers in rounded monocyte/macrophage cells that were abundant at the inner retinal surface, in the GCL and INL layers, and in the optic nerves of the spOHT-challenged eyes. Co-staining with the marker protein GFAP showed shows astrocytes (light grey, top panels). **C, D**. Control immunostaining in stOHT-challenged optic nerves showed no CD45^{+} monocytes in both retina and optic nerves. Small punctate CD45+CD11b+ cells are ramified microglia. Bright green puncta represent citrin-labeled ASC specks labeling of the mature inflammasome macro complexes that were abundant in the optic nerve astrocytes and in the infiltrating cells (yellow arrows). **E,F.** Control immunostaining in sham-operated controls showed only weakly labeled CD45+CD11b+ microglial cells in the IPL of the retina.

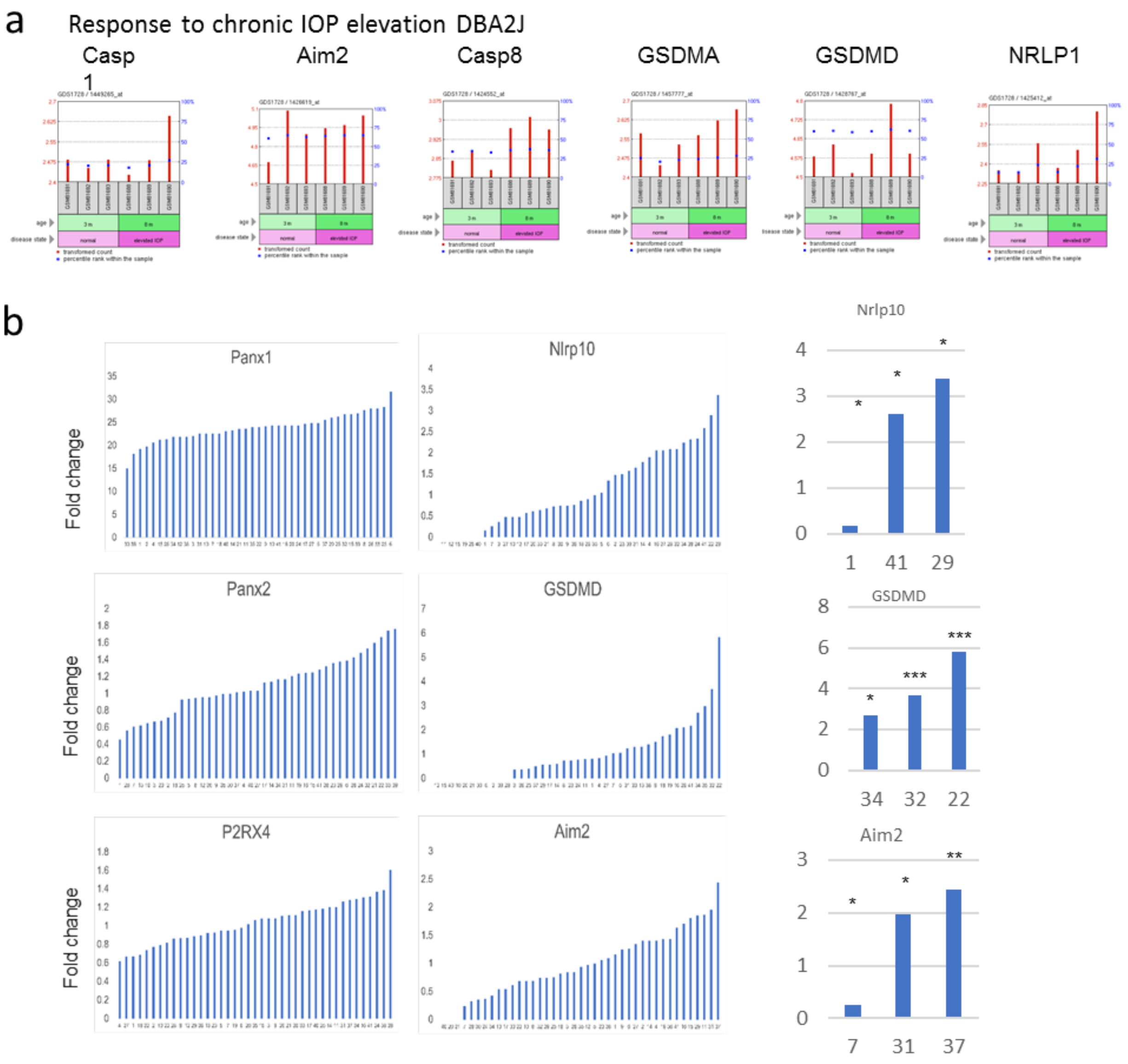

Supplement Figure S6. A. Meta-analysis of publicly available murine transcriptomic data (GEO GD51728), revealed evidence of inflammasome gene activation in aging DBA2J mice with glaucoma vs young pre-disease animals. **B.** Meta-analysis of murine scRNAseq dataset (https://www.ncbi.nlm.nih.gov/geo/query/acc.cgi?acc=GSE115404) showing differential activation of several upstream inflammasome regulator genes panx1, panx2, p2rx4; danger sensor genes NLRP10 and aim2, pore protein gene gsdmD reveal significant variability in these gene expression levels among different RGC subtypes.

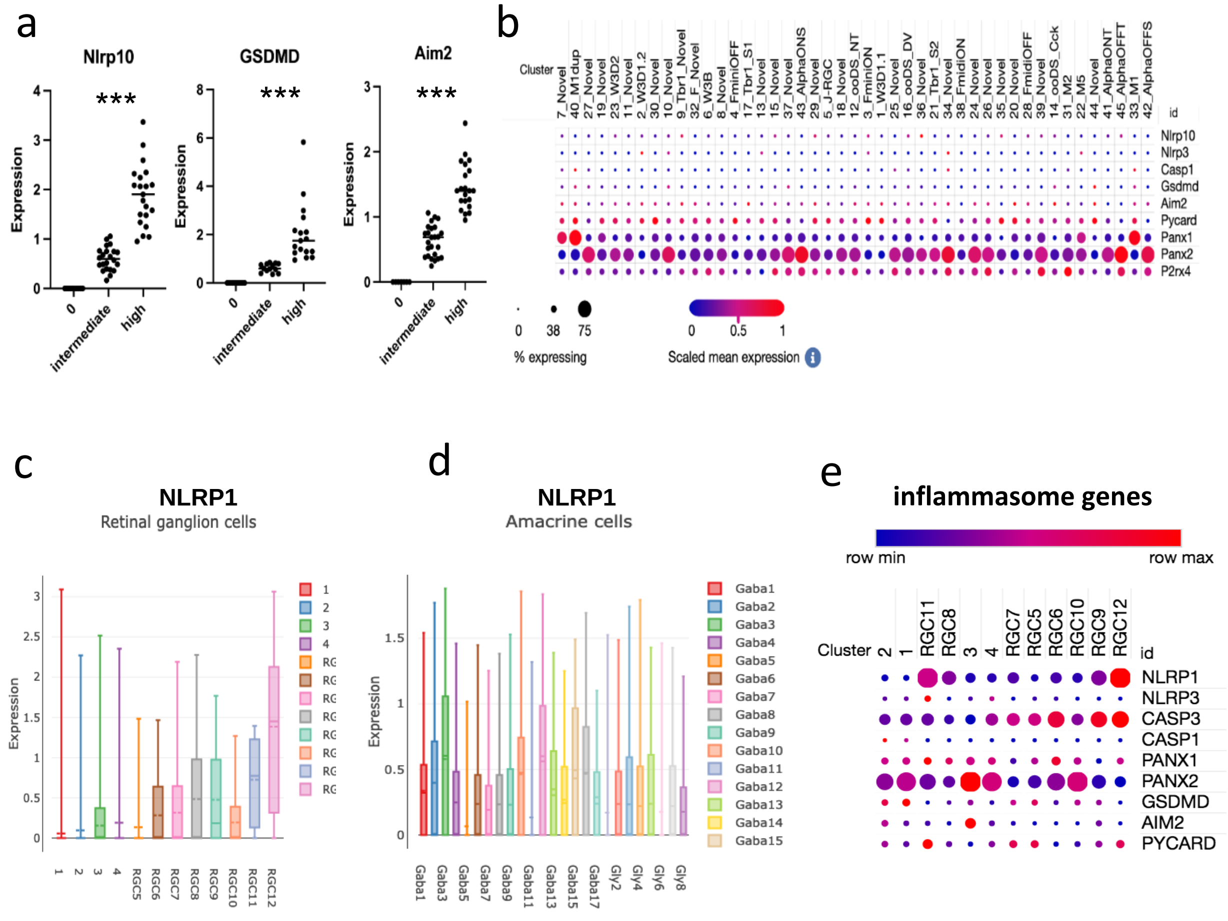

Supplement Figure S7. Meta-analysis of RGC subtype-specific scRNAseq data for inflammasome gene expression from human retina. A. Scatter plots for *NLRP10, AIM2,* and *GSDMD* expression in averaged low- and high-expressing RGC subtypes. **B** Relative expression of the inflammasome pathway genes among different RGC subtypes in the murine retina. **C-D** Relative expression of the *Nrlp1* gene among the most abundant RGC subtypes in the human fovea (Data source: https://singlecell.broadinstitute.org/ study #SCP839). **E.** Dot plot of the key inflammasome pathway genes among the most abundant RGC subtypes in the human fovea.

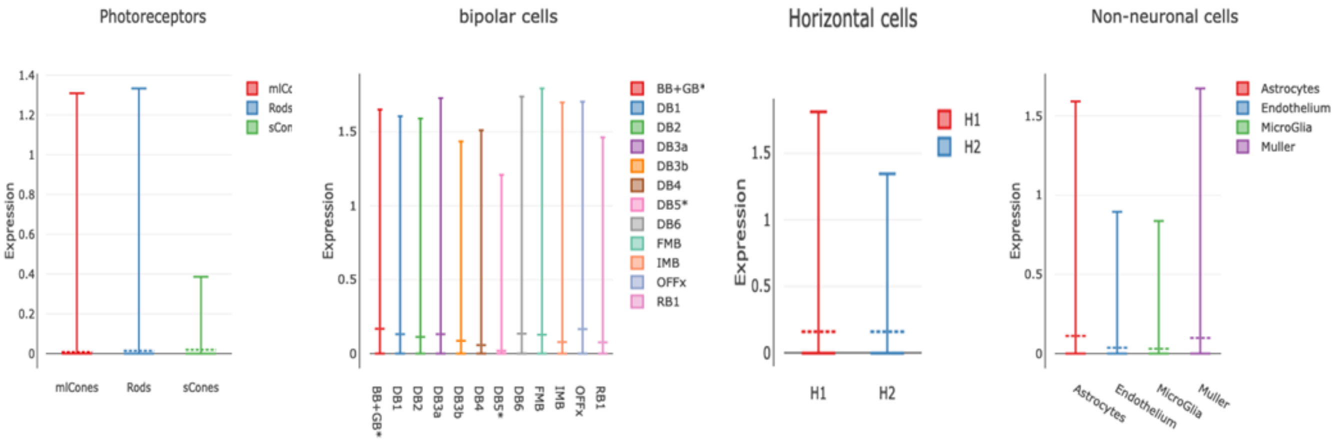

Supplement Figure S8. Meta-analysis of scRNAseq data from human photoreceptors, bipolar, horizontal, and non-neuronal cells for expression of the inflammasome-associated genes (Data source: https://singlecell.broadinstitute.org/ study #SCP839).
